# Portal venous contrast enhancement ratio of the adrenal glands and spleen as prognostic marker of mortality in patients with acute mesenteric ischemia

**DOI:** 10.1007/s00261-024-04247-2

**Published:** 2024-03-25

**Authors:** Felix Pfister, Matthias Mehdorn, Christoph Schwartner, Daniel Seehofer, Hans-Michael Tautenhahn, Manuel Florian Struck, Timm Denecke, Hans-Jonas Meyer

**Affiliations:** 1 Department of Diagnostic and Interventional Radiology, University of Leipzig, Leipzig, Germany; 2 Department of Visceral, Transplant, Thoracic and Vascular Surgery, University of Leipzig, Leipzig, Germany; 3 Department of Anesthesiology and Intensive Care Medicine, University Hospital Leipzig, Leipzig, Germany; 4 Department of Diagnostic and Interventional Radiology, University Hospital Leipzig, Liebigstr. 20, 04103 Leipzig, Germany

**Keywords:** Computed tomography, Acute mesenteric ischemia, Prognostic factor, Adrenal gland

## Abstract

**Purpose:**

Contrast enhancement of the adrenal gland defined by computed tomography (CT) was previously analyzed as a prognostic factor for critically ill patients in various diseases. However, no study investigated this quantitative parameter in patients with acute mesenteric ischemia. Therefore, the aim of this study was to evaluate the prognostic value of the contrast enhancement of the adrenal glands in patients with clinically suspected AMI.

**Methods:**

All patients with clinically suspected AMI were retrospectively assessed between 2016 and 2020. All patients underwent surgical exploration after CT imaging. Overall, 134 patients (52 female patients, 38.8%) with a mean age of 69.2 ± 12.4 years were included into the present analysis. For all patients, the preoperative CT was used to calculate the contrast media enhancement of the adrenal glands and the spleen.

**Results:**

A total of 27 patients (18.5%) died within the first 24 h and over the following 30-day 94 patients (68.6%) died. There were statistically significant differences regarding the mean values for adrenal-to-spleen ratio for 24-h mortality (*p* = 0.001) and 30-day mortality (*p* = 0.004), whereas the radiodensity of the inferior vena cava and the radiodensity of the spleen was statistically significant between survivors and non-survivors after 30 days (*p* = 0.037 and *p* = 0.028, respectively). In Cox regression analysis, mean adrenal radiodensity was associated with 24-h mortality (HR 1.09, 95% CI 1.02–1.16, *p* = 0.01) but not with 30-day mortality (HR 1.03, 95% CI 0.99–1.07, *p* = 0.13).

**Conclusion:**

The contrast media enhancement of the adrenal gland is associated with the 24-h and 30-day mortality in patients with AMI. However, the prognostic relevance for translation into clinical routine needs to be validated in other cohorts.

**Graphical abstract:**

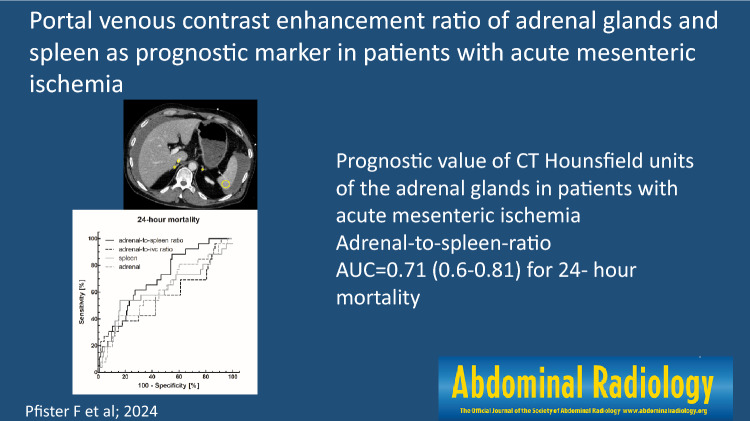

## Introduction

Acute mesenteric ischemia (AMI) is a potentially life-threatening medical condition but rather rare differential diagnosis in patients presenting with acute abdominal pain. Mesenteric ischemia is caused by insufficient blood supply typically to the small bowel [1].

In the acute setting, in addition to clinical examination and assessment of laboratory parameters, a contrast-enhanced computed tomography (CT) of the abdomen is recommended including a non-contrast, an arterial, and a portal venous phase [2]. Despite improvements in the imaging modalities correct diagnosis of acute mesenteric ischemia is still frequently delayed leading to a very high mortality of up to 30–70% of cases [3, 4].

An urgent laparoscopy or laparotomy is recommended in patients with signs of peritonitis to resect non-viable bowel segments if needed and to assure revascularization to preserve viable bowel segments in endovascular treatment [2].

In a variety of critically ill patients of the intensive care unit (ICU), Winzer et al. demonstrated that the portal venous adrenal-to-spleen ratio is a good predictor for mortality based on the CT hypoperfusions complex. This study population included 10.5% patients with acute mesenteric ischemia [5]. A higher mortality was seen in patients with a relative hyperperfusion of the adrenal glands in comparison with a hypoperfusion of the spleen. While hypoperfusion of the spleen was also correlated with higher mortality, the adrenal-to-spleen ratio in a portal venous phase showed the best discriminatory power to predict mortality [5]. Other studies have also demonstrated the potential benefit of this novel quantitative imaging marker in other populations with critically illness [6–9]. It could be crucial to provide prognostic quantitative imaging markers from CT images as it is routinely performed for diagnostic purposes in patients with AMI.

Thus, we sought to investigate a study population solely consisting of patients with AMI to confirm the prognostic relevance of these CT imaging markers.

The goal of this study was to examine whether the radiodensities of inferior vena cava (IVC), spleen, the adrenal glands, or especially the adrenal-to-spleen ratio would be viable parameters to predict mortality in patients with AMI.

## Methods

### Patients and study design

For this retrospective, observational study, all procedures performed involving human participants were in accordance with the ethical standards of the institutional and/or national research committee and with the 1964 Helsinki declaration and its later amendments or comparable ethical standards. It received ethical approval from the local ethics committee at the medical faculty of the university of Leipzig (EK: 165/23). Informed consent was waived due to the retrospective observation design of the present study. All data were handled in anonymized form and privacy issues were preserved.

Patients of our tertiary referral hospital with clinically suspected AMI were retrospectively assessed within the time period 2016 to 2020. We retrospectively analyzed CT scans of patients with clinically suspected mesenteric ischemia from our university hospital who had undergone a contrast-enhanced CT of the abdomen with at least a portal venous phase who subsequently underwent a laparoscopy/laparotomy for further exploration and/or treatment. Patients with insufficient follow-up data were excluded of this study. The investigated CT was performed within 24 h before the surgery, in most cases immediately before surgery. The serum markers were also obtained within 24 h prior to surgery. Patients were excluded, when imaging or clinical data were not within the reported time frame and CT imaging had been carried out without contrast media application.

### Clinical parameters

Etiology of AMI, analysis of co-morbidities in patient’s history included arterial hypertension, smoking, diabetes mellitus, dyslipidemia, and history of cardiovascular disorder. The following serum markers were obtained from venous blood at least within 24 h before the surgery: White blood cell count (GPT), lactate (mmol/L), C-reactive protein (mg/dl), hemoglobin (g/L), and lactate dehydrogenase (U/L). Mortality was assessed in days after surgical exploration. All-cause mortality was assessed over a time period of 30 days and 24 h.

### Surgical exploration and bowel segment resection

All patients underwent surgical exploration either by laparoscopy or laparotomy after emergency indication. Bowel segments suspect of ischemia were identified and the need for intestinal resection obtained from the surgical report.

### CT acquisition

The patients were examined using a 128-slice CT scanner (Ingenuity, Philips, Amsterdam, the Netherlands) during clinical routine. Intravenous administration of an iodine-based contrast medium (90-mL Imeron 400 MCT, Bracco Imaging Germany GmbH, Konstanz, Germany) was given at a rate of 4.0 mL/s via a peripheral venous line. Automatic bolus tracking was performed in the descending aorta with a trigger of 100 Hounsfield units (HU). CT was performed in arterial and portal venous phase in every case. Typical imaging parameters were as follows: 100 kVp; 125 mAs; slice thickness, 1 mm; and pitch, 0.9.

### Image analysis

Evaluation of image data was performed on a PACS workstation (iDS7, Sectra AB, Linköping, Sweden) using Region of interest (ROI) measurements to analyze the attenuation of the IVC, both adrenal glands and the spleen in the portal venous phase on images with the minimal slice thickness (1 and 3 mm). In accordance with previous studies, the attenuation of the spleen was measured at three axial levels (each ROI 2.0 cm^2^ in size) and the adrenal attenuation was measured preferably in the confluence of both adrenal limbs with a preferred ROI size of 30–40 mm^2^. Attenuation of the IVC was measured at the axial level of the right adrenal gland. Infarct-like triangular hypodense areas at the splenic periphery were excluded from the measurement. The adrenal-to-IVC ratio was defined as the mean adrenal radiodensities divided by the IVC’s radiodensity. The mean adrenal radiodensities were divided by the averaged radiodensities of the spleen to get the adrenal-to-spleen ratio [2]. The first reading was performed by a resident with 3 years of general radiology experience. Another reading was performed by a senior radiologist with 9 years of experience in gastrointestinal radiology.

**Fig. 1:**
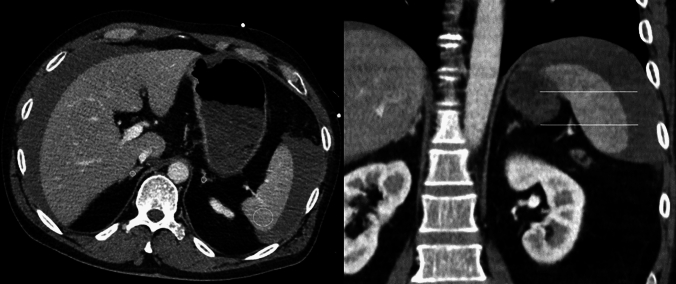
Representative patient of our patient sample with an axial and coronal CT image in portal venous contrast phase. The region of interest placement is drawn within the boundaries of the adrenal glands and the spleen to measure the radiodensities. Free fluid surrounding the liver and spleen can also be appreciated

Figure 1 provides a representative case of our patient sample for illustrative purposes.

### Statistical analysis

Statistical analysis was performed using GraphPad PRISM Ver. 5.01 (GraphPad Software, Boston, USA) and SPSS Ver. 29 (IBM, New York, USA). The study population characteristics were given as mean and standard deviation for continuous variables. Regarding 24-h, 30-day, and overall mortality as well as need for resection, we performed a Mann–Whitney *U* test and a subsequent multivariate Cox regression analysis adjusting for sex, hypertension, dyslipidemia, history of cardiovascular disease, and smoker status. To evaluate the suitability of the measured radiodensities and the radiodensity ratios for predicting mortality and need for resection, we performed a receiver operating curve (ROC) analysis and calculated threshold values. As a measure for diagnostic accuracy, the area under the curve (AUC) was used [10]. Regarding its discriminatory power AUC was considered as not useful < 0.5, as bad > 0.5–0.6, as sufficient > 0.6–0.7, as good > 0.7–0.8, as very good > 0.8–0.9, and as excellent > 0.9–1. Interreader variability was assessed using intraclass correlation coefficient (ICC) with confidence intervals (CI).

In all instances, *p* values below 0.05 were considered as statistically significant.

## Results

27 patients (18.5%) died within the first 24 h and over the following 30-day, 94 patients (68.6%) died. An overview of the investigated patient sample is provided in Table 1.

**Table 1:** Demographic characteristics of the patient sample according to 24-h and 30-day mortality

Parameter	Non-survivors 24 h (*n* = 27)	Survivors 24 h (*n* = 110)	*p* value	Non-survivors 30 days (*n* = 94)	Survivors 30 days (*n* = 43)	*p* value
Age (years)	72.4 ± 9.7	68.5 ± 12.9	0.202	70.9 ± 10.3	65.7 ± 15.6	0.163
Sex (female, *n*, %)	9 (33.3)	43 (39.1)	0.66*	40 (42.6)	12 (27.9)	0.13*
BMI (kg/m^2^)	28.8 ± 6.7	28.1 ± 9.1	0.259	28.3 ± 8.2	28.3 ± 9.7	0.917
History of hypertension (*n*, %)	20 (74.1)	80 (72.7)	1,00*	72 (76.6)	28 (65.1)	0.21*
History of dyslipidemia (*n*, %)	13 (48.1)	48 (43.6)	0.67*	41 (42.6)	20 (46.5)	0.85*
History of cardiovascular disease (*n*, %)	17 (63.0)	73 (566.4)	0.65*	64 (68.1)	26 (60.4)	0.69*
Diabetes mellitus (*n*, %)	11 (40.7)	36 (32.7)	0.50*	34 (36.2)	13 (30.2)	0.56*
Smoker (*n*, %)	2 (7.4)	22 (20.0)	0.16*	17 (18.1)	7 (16.3)	1.00*
Bowel resection (*n*, %)	23 (85.2)	88 (80.0)	0.78*	80 (86.0)	31 (72.1)	0.06*
C-reactive protein (mg/L)	137.2 ± 146	154.8 ± 143.9	0.608	187.3 ± 148.8	112.3 ± 127.4	**0.037**
Lactate (mmol/L)	11.3 ± 4.6	4.5 ± 4.0	** < 0.001**	7.1 ± 5.3	2.8 ± 1.9	** < 0.001**
Hemoglobin (mmol/L)	8.8 ± 2.7	10.1 ± 3.2	0.053	8.9 ± 2.7	11.9 ± 3.1	** < 0.001**
Lactate dehydrogenase (U/L)	10.1 ± 3.8	5.9 ± 3.6	** < 0.001**	7.3 ± 3.4	5.9 ± 2.8	0.080
White blood cell count (G/L)	21.2 ± 13.6	17.5 ± 9.6	0.220	19.5 ± 10.9	15.5 ± 9.4	**0.030**
IVC (HU)	135.1 ± 32.5	133.0 ± 32.5	0.694	137.1 ± 34.1	125.1 ± 26.6	**0.032**
Adrenal (HU)	104.7 ± 38.4	92.1 ± 29.1	0.123	97.2 ± 34.0	88.9 ± 24.3	0.235
Spleen (HU)	85.0 ± 34.1	94.9 ± 25.1	0.059	89.6 ± 27.9	100.5 ± 24.6	**0.028**
Adrenal-to-IVC ratio	0.79 ± 0.28	0.70 ± 0.19	0.478	0.72 ± 0.22	0.72 ± 0.19	0.973
Adrenal-to-spleen ratio	1.39 ± 0.67	1.02 ± 0.46	**0.001**	1.18 ± 0.59	0.91 ± 0.28	**0.004**

Statistically significant values are highlighted in bold

*BMI* body mass index, *HU* Hounsfield unit, *IVC* inferior vena cava

*Indicates *p* values provided by Fisher’s exact test

During the surgical exploration, overall, 112 patients (76.7%) underwent ischemic bowel segment resection.

### Interreader variability

The investigated CT parameters showed a low interreader variability with reported ICC from 0.41 for the adrenal/IVC ratio (95% CI 0.10–0.64) up to 0.97 (95% CI 0.96–0.99) for the spleen radiodensity. The results of the interreader variability are summarized in Table 2.

**Table 2:** Interreader variability of the investigated CT parameters

Parameter	ICC	95% CI
IVC radiodensity	0.83	0.7–0.9
Adrenal gland right radiodensity	0.92	0.86–0.95
Adrenal gland left radiodensity	0.92	0.85–0.95
Adrenal radiodensity	0.74	0.54–0.85
Spleen radiodensity	0.97	0.96–0.99
Adrenal-to-IVC ratio	0.41	0.10–0.64
Adrenal-to-spleen ratio	0.76	0.56–0.87

*ICC* intraclass coefficient, *CI* confidence interval

### Discrimination analysis

Using the Mann–Whitney *U* test there was a significant difference in means for the adrenal-to-spleen ratio regarding 24-h mortality (*p* = 0.001) and 30-day mortality (*p* = 0.004). The radiodensity of the inferior vena cava showed significant difference in means for 30-day mortality (*p* = 0.037) and mean radiodensity of the spleen were significant for 30-day mortality (*p* = 0.028) (Table 1).

Apart from the hypoperfusion parameters mean levels of venous lactate were significantly higher for both 24-h and 30-day mortality. Serum-level of C-reactive-protein was significantly higher (*p* = 0.002) and history of cardiovascular disease was significantly more frequent (*p* = 0.023) in patients in which ischemic bowel segments were resected (Table 3). However, there was no significant difference regarding need for bowel resection for any other parameter including the measured radiodensities.

**Table 3:** Demographic characteristics of the patient sample according to surgical resection

Parameter	Resection (*n* = 112)	No resection (*n* = 25)	*p* value
Age (years)	69.51 (± 12.47)	68.04 (± 12.39)	0.396
Sex (female, * n*, %)	42 (37.5)	10 (40)	0.823*
BMI (kg/m^2^)	27.71 (± 7.95)	30.80 (± 11.12)	0.073
History of hypertension (*n*, %)	77 (68.75)	23 (92)	0.023*
History of dyslipidemia (*n*, %)	50 (44.64)	11 (44)	1*
History of cardiovascular disease (*n*, %)	72 (64.29)	18 (72)	0.641*
Diabetes mellitus (*n*, %)	37 (33.04)	10 (40)	0.496*
Smoker (*n*, %)	22 (19.64)	2 (8)	0.246*
C-reactive protein (mg/L)	173.58 (± 142.96)	62.98 (± 109.19)	**0.002**
Lactate (mmol/L)	5.77 (± 4.70)	6.22 (± 5.97)	0.547
Hemoglobin (mmol/L)	9.81 (± 3.02)	10.09 (± 3.64)	0.77
Lactate dehydrogenase (U/L)	6.70 (± 3.28)	6.33 (± 2.93)	0.795
White blood cell count (G/L)	18.32 (± 10.28)	17.83 (± 12.12)	0.585
IVC (HU)	134.17 (± 32.09)	129.85 (± 34.16)	0.396
Adrenal (HU)	96.75 (± 32.51)	84.89 (± 23.98)	0.098
Spleen (HU)	94.02 (± 26.45)	88.19 (± 30.88)	0.332
Adrenal-to-IVC ratio	0.73 (± 0.22)	0.66 (± 0.1)	0.086
Adrenal-to-spleen ratio	1.09 (± 0.49)	1.11 (± 0.68)	0.623

Statistically significant values are highlighted in bold

*BMI* body mass index, *HU* Hounsfield unit, *IVC* inferior vena cava

*Indicates *p* values provided by Fisher’s exact test

### Cox regression analysis

For 24-h mortality, the multivariate Cox regression showed a significant hazard ratio (HR) of 1.09 (95% CI 1.02–1.16, *p* = 0.01) for mean adrenal radiodensities (Table 4). Furthermore, significant effects were observed for serum lactate HR 1.32, CI 1.15–1.50, *p* < 0.001) and sex (HR 7.52, 95% CI 1.28–44.04, *p* = 0.025), whereas no further differences were shown for the other investigated hypoperfusion parameters of the IVC, adrenal glands, or spleen. Regarding 30-day mortality, the only significant difference was found for elevated serum lactate levels with a hazard ratio of 1.15 (CI 1.08–1.22) and a *p* < 0.001 (Table 5).

**Table 4:** Cox regression analysis for 24-h mortality

Parameter	Hazard ratio (95% CI)	*p* value
Age	1.04 (0.98–1.10)	0.199
Sex (male)	7.52 (1.28–44.04)	**0.025**
BMI	1.07 (0.96–1.20)	0.222
Hypertension	1.58 (0.41–6.17)	0.509
Dyslipidemia	1.02 (0.34–3.07)	0.966
Diabetes	0.34 (0.09–1.27)	0.162
Smoking	1.05 (0.15–7.18)	0.965
Bowel resection	1.34 (0.24–7.56)	0.738
Serum lactate	1.31 (1.15–1.50)	**< 0.001**
IVC	0.95 (0.90–1.01)	0.08
Adrenal radiodensity	1.09 (1.02–1.16)	**0.011**
Spleen radiodensity	0.99 (0.93–1.05)	0.685
Adrenal-to-IVC ratio	0.001 (< 0.01–4.40)	0.108
Adrenal-to-spleen ratio	0.51 (0.03–8.97)	0.647

Statistically significant values are highlighted in bold

*BMI* body mass index, *IVC* inferior vena cava, *CI* confidence interval

**Table 5:** Cox regression analysis for 30-day mortality

Parameter	Hazard ratio (95% CI)	*p* value
Age	1.02 (1.00–1.05)	0.054
Sex (male)	1.29 (0.52–3.23)	0.584
BMI	1.02 (0.97–1.08)	0.379
Hypertension	0.95 (0.49–1.83)	0.878
Dyslipidemia	0.92 (0.53–1.59)	0.769
Diabetes	0.66 (0.37–1.18)	0.162
Smoking	1.35 (0.69–2.66)	0.382
Bowel resection	1.44(0.68–3.05)	0.345
Serum lactate	1.15 (1.08–1.22)	**< 0.001**
IVC	0.99 (0.97–1.02)	0.635
Adrenal	1.03 (0.99–1.07)	0.136
Spleen	0.97 (0.95–1.00)	0.079
Adrenal-to-IVC ratio	0.32 (0.01–16.47)	0.576
Adrenal-to-spleen ratio	0.34 (0.09–1.32)	0.120

Statistically significant values are highlighted in bold

*BMI* body mass index, *IVC* inferior vena cava, *CI* confidence interval

### ROC analysis

**Fig. 2:**
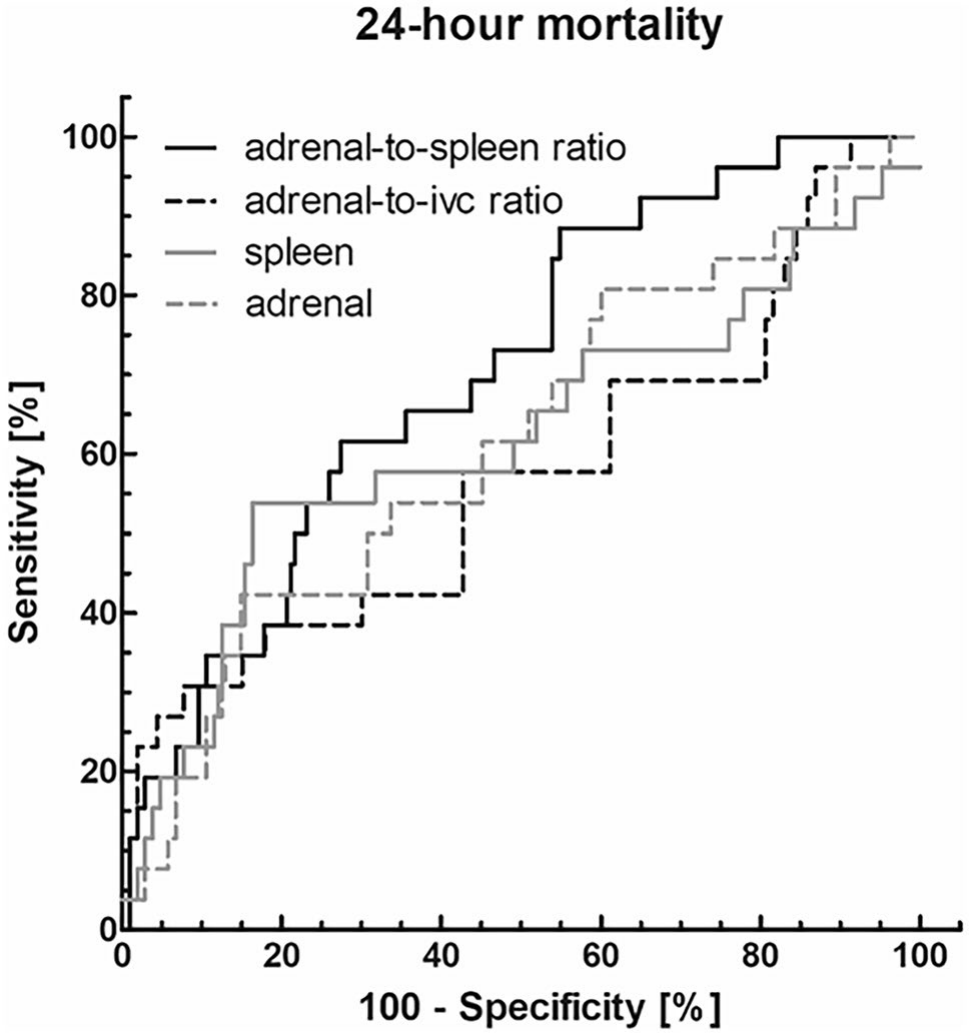
ROC analysis to predict 24-h mortality. The adrenal-to-spleen ratio showed a good discriminative power with an AUC = 0.71 (CI 0.60–0.81). A cut-off value of 1.103 leads to a sensitivity of 61.5% and a specificity of 73.1%

The adrenal-to-spleen ratio showed a good discriminative power for predicting patients’ mortality with an AUC = 0.71 (CI 0.60–0.81) for 24-h mortality. A cut-off value of 1.103 leads to a sensitivity of 61.5% and a specificity of 73.1%. Sufficient discriminative power was demonstrated for mean adrenal radiodensities with an AUC of 0.61 (CI 0.49–0.74) (Fig. 2).

**Fig. 3:**
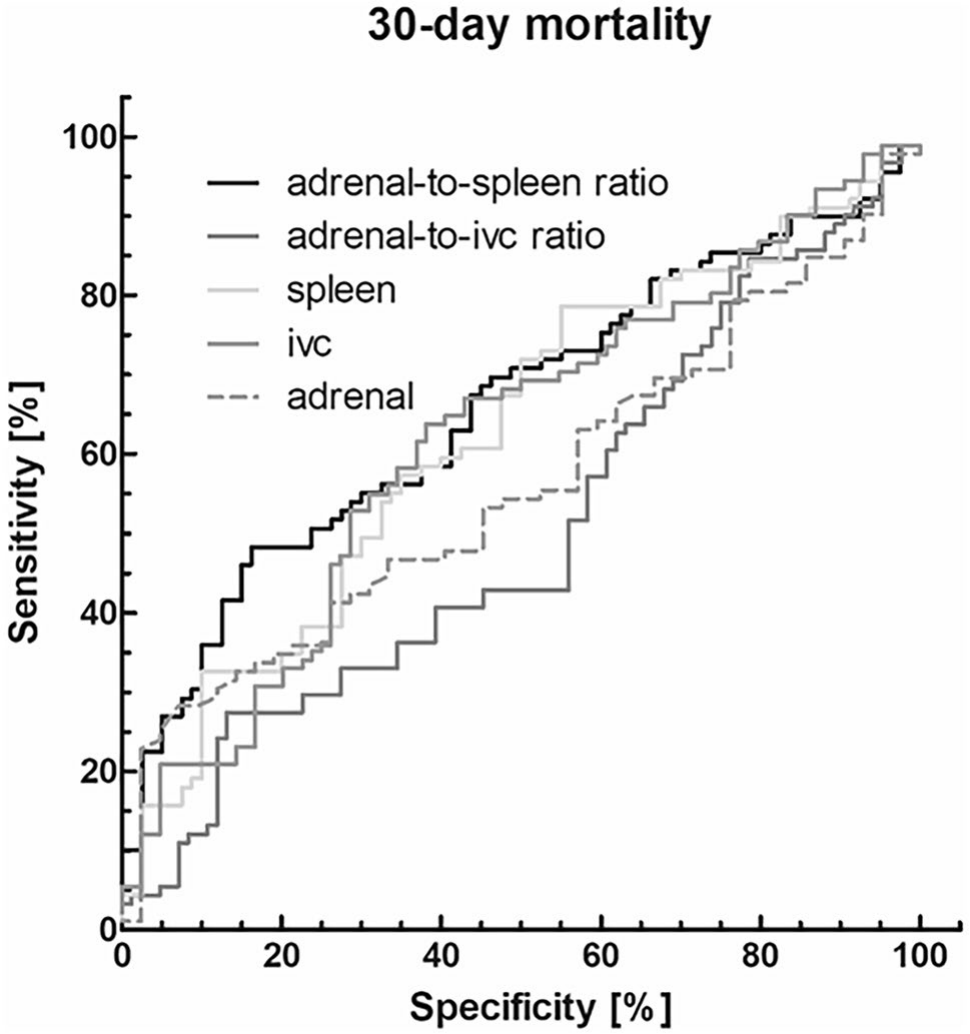
ROC analysis to predict 30-day mortality. Adrenal-to-spleen ratio showed a good discriminative power with an AUC of 0.66 (CI 0.56–0.75). The cut-off value of 0.904 reached a sensitivity of 64.4% and a specificity of 57.5%

Regarding 30-day mortality, sufficient discriminative power could be shown for adrenal-to-spleen ratio with an AUC of 0.66 (CI 0.56–0.75) and for IVC radiodensities with an AUC of 0.60 (CI 0.50–0.71) (Fig. 3). However, a cut-off value of 0.904 reached a sensitivity of 64.4% and a similar specificity of 57.5%. Mean adrenal and splenic radiodensities as well as adrenal-to-IVC ratio showed either bad or no power to predict mortality over 30 days.

Using the proposed cut-off value of 1.41 by Winzer et al. sensitivity was 34.6% and specificity was 86.5% for 24-h mortality. For 30-day mortality, only a sensitivity of 23.3% with a specificity of 97.5% was achieved.

## Discussion

Our study aimed to evaluate whether quantitative imaging parameters obtained from a preoperative CT-scan could be used to predict mortality in patients with suspected AMI.

According to the literature the imaging finding with prognostic relevance is pneumatosis intestinalis with a reported odds ratio of 2.86 for short-term mortality [11]. In another study investigating prognostic CT findings, only the concomitant presence of pneumatosis and porto-mesenteric venous gas showed an odds ratio of 1.95 (95%CI 0.49–7.79, *p* = 0.34) for the occurrence of transmural necrosis [12]. Yet, other CT findings such as mural thickening, bowel distension, pneumatosis alone, and presence of thrombi or emboli were not different in patients with and without transmural necrosis [12].

This highlights the importance of new CT imaging markers to stratify patients at risk.

Another recent study investigated the prognostic relevance of CT-defined body composition parameters in patients with AMI to extract novel prognostic markers from the CT images [13]. This is especially of importance as even established laboratory parameters comprising lactate, D-dimers, C-reactive protein, and white blood cell count have low accuracy to predict a poor clinical outcome in these patients [14].

That is why there is definite need for further quantitative parameters to correctly predict a poor clinical outcome in patients with AMI. This would be crucial as the investigated radiodensities parameters in the current study were already demonstrated to be of prognostic relevance in critically ill patients [5–7].

In a patient collective with various underlying pathologies, Winzer et al. found the adrenal-to-spleen ratio of a portal venous scan to be a good predictor for 72-h mortality with an AUC of 0.94 using a cut-off value of > 1.4 [5]. This resulted in a specificity above 98% and a sensitivity at around 80% in a patient cohort of 203 patients [5]. The authors included different underlying causes of critical illness, most commonly with postoperative complications (128 patients) and septic shock (57 patients). Notably, the mortality was significantly lower in the patient cohort by Winzer et al. with 9.9% after 24 h and 24.1% after 72 h compared with the present study [5]. This might explain the divergent results regarding the accuracies.

In the present patient collective of patients with AMI, we found significant difference in means of the adrenal-to-spleen ratio especially regarding 24 h- and 30-day mortality. However, the diagnostic accuracy remained low with a maximum of 0.70 for 24-h mortality. Using the same cut-off value of > 1.41 as proposed by Winzer et al. [5] only a sensitivity of 34.6% and specificity of 86.5% could be achieved. Even with a more moderate cut-off value of > 1.103 achievable sensitivity and specificity remained poor with 61.5% and 73.1%, respectively.

Presumably, the identified differences of the investigated CT radiodensities could be explained by differences in the clinical conditions and the shock state of the investigated patient cohorts. The underlying causes of critical illness were more heterogeneous in the patient sample of Winzer et al. [5].

Hyperattenuation of the adrenal glands and hypoperfusion (and therefore hypoattenuation) of the spleen are part of the CT hypoperfusion complex which consists of a multitude of signs related to shock and shock-related hypotension and were reported to be of diagnostic and prognostic importance [9, 15–17].

Since the patients in our collective received the CT-scan in an acute setting, patients might have been less critically ill, and the scan was possibly performed shorter after the onset of symptoms and therefore less likely to have developed shock and sufficient changes in attenuation of the adrenal glands and spleen compared to the patient cohort of Winzer et al.

In contrast to the above-mentioned studies, one study found the absence of hyperattenuation of the adrenal glands to be related to mortality over 28 days for critically ill patients in septic shock [8]. This might indicate that also less contrast enhancement of the adrenal glands is in some cases a sign for worse prognosis.

Another reason for the identified results might be that the perfusion of the adrenal glands is reduced due to vessel stenosis, a common precondition of patients with AMI. In the study by Winzer et al. patients with stenosis of the celiac trunk were excluded from the analysis [5].

The prognostic relevance of adrenal gland enhancement was also evaluated in other diseases. Schek et al. [18] investigated 292 polytrauma patients in a retrospective analysis. Of these, 18 patients (6.1%) showed a stronger enhancement of the adrenal gland compared to the IVC, which was a statistically significant predictor for poor clinical outcome. Moreover, mean adrenal enhancement was significantly higher in non-survivors compared with survivors (101.9 ± 40.6 HU vs. 86.1 ± 27.0 HU; *p* < 0.001).

Boos et al. investigated 88 patients of the ICU with clinical deterioration. 43.2% of these patients showed a high adrenal gland enhancement, which was also a predictor for poor outcome. Of this patient cohort, overall, 50% died, whereas only 16% died of the patient group with regular adrenal gland enhancement [19].

In another study investigating 71 adult patients with hypovolemic shock found a statistically significant reduction, not only of splenic volumes, but also in splenic attenuation values compared with a control population (105 ± 34 HU vs. 134 ± 25 HU; *p* < 0.001) [20]. Despite these promising results of the prognostic relevance of adrenal gland enhancement, there is still need for further investigation for clinical translation.

The present study has limitations. First, it is a retrospective single-center study with known inherent bias. Second, despite the quantitative nature of the measurements, there can still be some interreader variability. However, in our analysis, the interreader variability was low, which is consistent with a previous study [7]. Third, there might be some dependency of the adrenal gland enhancement on contrast media type, concentration, and flow, which reduces possible external validity. Forth, it is important to discuss the selection of only surgical patients in the present analysis. The generalization of our results to all patients with AMI remains limited, as it cannot be adjusted for patients with less severity, which could be treated conservatively. Fifth, co-morbidities, such as congestive heart failure or sepsis, could have a confounding role on the investigated radiodensities. Due to the study design, there was not enough data to adjust for these co-morbidities. There is also the potential heterogeneity of contrast enhancement of the spleen especially in earlier contrast media phases.

In conclusion, the contrast media enhancement of the adrenal gland is associated with the 24-h and 30-day mortality in patients with AMI. However, the prognostic relevance needs to be validated in other cohorts for a possible translation into clinical routine.
